# Relationship between sortilin with ApoB/ApoA ratio in military personnel with arterial hypertension

**DOI:** 10.1038/s41598-026-51862-8

**Published:** 2026-06-22

**Authors:** Ilaha Abdurahmanova, Sona Gahramanova, Vesadet Azizov, Esmira M. Nasibova

**Affiliations:** 1 Department of Military Medicine, Medical Center of the Ministry of Emergency Situations of the Republic of Azerbaijan, Military Research Institute of the National Defense University, Baku, Azerbaijan; 2 Department of Anesthesiology, Reanimatology and Intensive Care, Azerbaijan Medical University, Baku, Azerbaijan

**Keywords:** Biomarkers, Cardiology, Diseases, Medical research, Risk factors

## Abstract

The study investigates the relationship between sortilin levels and the ApoB/ApoA ratio in military personnel with arterial hypertension. A cross-sectional analytical study involving 120 male military personnel was conducted. Biochemical, hematological, and ELISA measurements were performed. Sortilin, ApoB, ApoB/ApoA ratio, and hs-CRP levels were significantly higher in the hypertensive group and correlated with systolic blood pressure. ROC analysis confirmed their discriminatory performance. Sortilin, ApoB, and hs-CRP are associated with hypertension and may be considered potential markers related to cardiovascular risk. However, due to the cross-sectional design, causal relationships cannot be established, and further large-scale prospective studies are required.

## Introduction

Arterial hypertension (AH) is one of the most common risk factors for the development of cardiovascular diseases and premature death worldwide^1–3^. In recent decades, the attention of researchers has been drawn to its manifestation and development in young people, including military personnel, who are often exposed to stress, high physical activity and specific service conditions^4,5^. In this context, it is important to understand how AH in young men can be associated with changes in the lipid profile and inflammatory processes in the body. The lipid profile, including cholesterol levels (including total, LDL, HDL and triglycerides), has a significant impact on the state of the vascular endothelium and the development of atherosclerosis, which is a key link in the pathogenesis of arterial hypertension^6,7^. In turn, chronic inflammatory processes play an important role in the activation of endothelial dysfunctions and the progression of hypertension^8–10^. This problem is especially acute in young military personnel, as they are exposed to physical overload, prolonged stress and specific psychological stress, which can be catalysts for the development of hypertension and its complications. The higher than expected prevalence of hypertension, even in occupational groups leading an active lifestyle, such as military personnel, contradicts recent studies reporting that physical activity is associated with a reduced risk of arterial hypertension (AH). This situation increases the need for a reliable biological marker that can be a precursor to hypertension and clarify its pathophysiology, in addition to classical methods.

### Aim of the work

The objective of this study was to investigate, to assess associations, the relationship between sortilin levels with the ApoB/ApoA ratio in hypertension detected in military personnel serving in the armed forces of Azerbaijan.

## Materials and methods

### Ethics statement

All methods were carried out in accordance with relevant guidelines and regulations. Ethical approval for this study was obtained from the Ethics Committee of the Azerbaijan Medical University (Protocol No. 13, dated 15.06.2022).

### Study design

The study is a single-center, observational, cross-sectional analytical study. In order to achieve the planned sample size, approximately 220 male military personnel who applied to the Medical Center of the Ministry of Emergency Situations of the Republic of Azerbaijan for a follow-up examination. After the participants were informed about the study, consent forms were obtained. After recording the demographic characteristics of the participants, visual and physical examinations were performed. Blood pressure values were measured in systolic and diastolic modes using the same tonometer (Bosh + Sohn, Germany). Biochemical studies (cholesterol, triglycerides, LDL, VLDL) and hemogram were performed using these blood samples. After laboratory testing, excess serum was stored at − 80 °C. These sera were stored until the day of testing for ApoB, ApoA, hs-CRP, and sortilin.

As a result of the examination, 55 male servicemen with arterial hypertension and 65 healthy male servicemen were consecutively enrolled. The group of patients who were diagnosed with hypertension was selected from among the patients who had hypertension as a primary pathology. Subjects with secondary hypertension, subjects with comorbidities in addition to hypertension (such as cardiovascular, renal and liver diseases), subjects with cancer (myelodysplastic syndrome, leukemia and lymphoma arising in the blood, bone marrow and lymph nodes), rheumatic diseases affecting the levels of inflammatory markers (such as IL-6, TNF-α and hs-CRP) (such as rheumatoid arthritis, SLE, ankylosing spondylitis, acute rheumatic fever), autoimmune diseases (such as Sjogren’s disease, Hashimoto’s thyroiditis, celiac disease, multiple sclerosis, inflammatory bowel disease), subjects with infection or inflammation, and subjects taking anti-inflammatory or antihyperlipidemic drugs were excluded from the study.

### Biochemical and hematological measurements

Hemogram measurements were performed on EDTA blood of all participants using an automatic hematology analyzer (Mindray BC-600, China). In addition, high-sensitivity C-reactive protein (hs-CRP), ApoA, ApoB, cholesterol, LDL and VLDL levels were measured in blood collected in plain tubes using a biochemistry autoanalyzer (Roche Cobas C 702, Mannheim, Germany). Inappropriate sample collections, clotted and excessively hemolyzed samples were not included in the study. Quality control studies were completed before all measurements.

### ELISA measurements

BT lab brand ELISA Kits (Shangai, China) were used to measure sortilin levels. The sensitivity of the kit was 0.94 ng/L and the measurement range was 2–600 ng/L, respectively. The intra-study coefficient of variation was < 10%. Measurements in the study were performed using BIO-TEK ELx500 ELISA Reader and BIO-TEK ELx50 (USA) wash devices.

### Statistical analysis

SPSS Statistic Software version 25 (IBM Corp, Chicago, USA) was used for the analysis of statistical data of this study. Statistical data were collected in Microsoft Excel 2010 Office program. Bar plots were used for graphical representation of nonparametric data. Chi-square test was used for evaluation of categorical data when comparing differences between groups. Kolmogorov–Smirnov test was used to evaluate whether the study data were normally distributed. Student t test was applied for parametric data and Mann–Whitney U test was applied for nonparametric data in comparisons of independent groups consisting of two groups. Spearman and Pearson correlation test were used for nonparametric data and parametric data, respectively, to determine correlations between independent variables. Receiver operating characteristic (ROC) analysis was used for the discriminatory ability of ApoB, ApoB/ApoA, hs-CRP, sortilin tests in determining HT. AUC value was accepted as a practical measure of test performance in determining the classification performance of the tests. For the minimum acceptable discrimination power, an AUC value of > 0.70 was accepted. If the AUC was between 0.8 and 0.9, the test was assumed to have high discrimination ability, and if AUC was > 0.9, it was assumed to have very high (excellent) discrimination ability.

## Results

### Comparison of demographic and clinical features

**Fig. 1:**
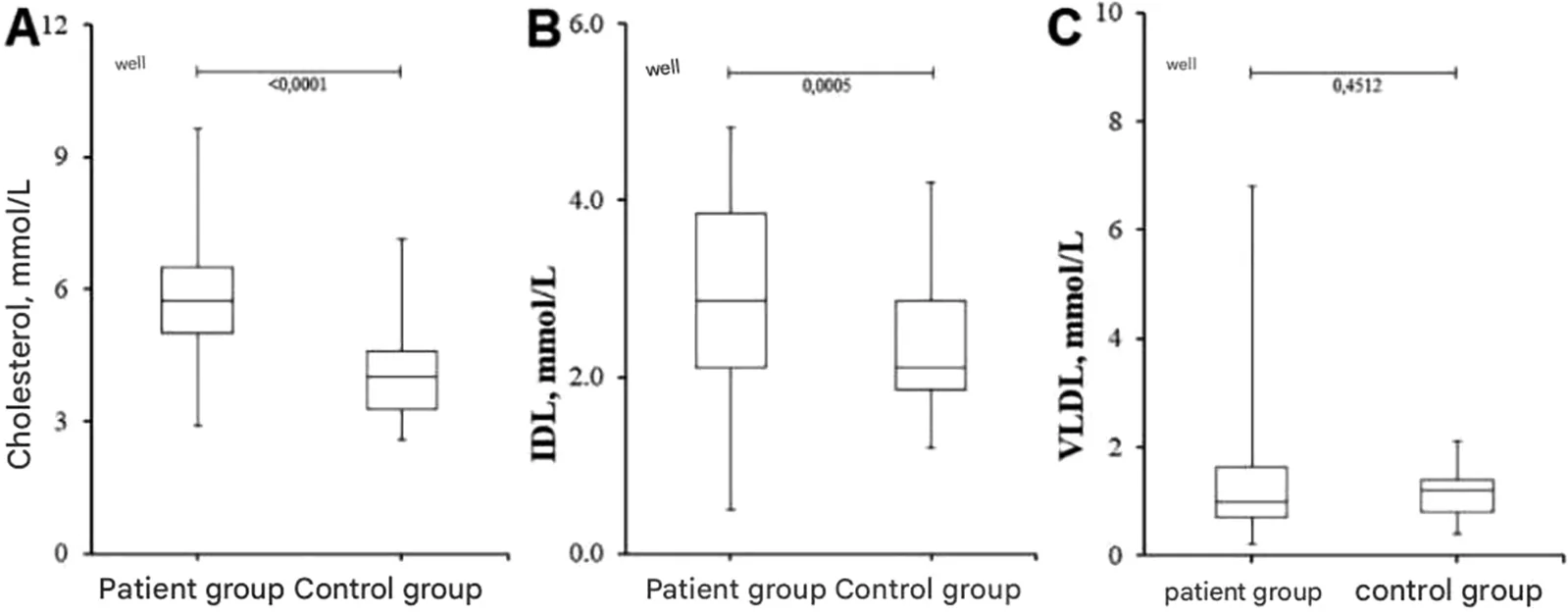
Box-plot graph representing cholesterol, LDL and VLDL levels of normal and hypertensive patient groups. Cholesterol and LDL levels are seen to be higher in the hypertensive patient group. **A** Mann Whitney U test. **B** Shows the comparison of ApoB/ApoA ratio values between hypertensive patients and the control group. **C** Demonstrates the correlation analysis between serum sortilin levels and ApoB/ApoA ratio in military personnel with arterial hypertension.

As a result of the study, the mean ages of the control (M/F: 65/0) and hypertensive patient (M/F: 55/0) groups were 36 ± 8 (23–46) and 37 ± 8 (23–45) years, respectively; mean BMI values were 23.5 ± 2.4 (17.8–29.8) and 23.6 ± 2.6 (16.8–30.8) kg/m^2^, respectively (Table 1). There was no difference between the groups formed from the male population in terms of age and BMI (*p* > 0.05) (Fig. 1). Therefore, the differences we detected between the groups in terms of other parameters were accepted to be independent of these three demographic features.

**Table 1:** Comparison of demographic characteristics and blood pressure values of the study groups.

	All groups	HT patient group (A)	Control group (B)	*p* value
n, E	120	55	65	–
Gender, M (%)	120 (%100)	55 (%100)	65 (%100)	ø
Age, years	36 ± 8	37 ± 8	36 ± 8	0.6235^b^
BMI, kg/m^2^	23.5 ± 2.5	23.6 ± 2.6	23.5 ± 2.4	0.7705^b^
Waist circumference, sm	80 ± 8.0	82 ± 7.7	78 ± 7.7	0.0029^b^*
SBP, mm/Hg	129 ± 22 128 (100–170)	150 ± 9 149 (124–170)	111 ± 10 107 (100–133)	< 0.0001^a^*
DBP, mm/Hg	69 ± 9 68 (48–90)	69 ± 9 68 (50–87)	69 ± 9 69 (48–90	0.3633^a^

When the study groups were examined in terms of waist circumference (WC), WC was found to be higher in the HT patient group (*p* = 0.0029) (Table 1).

### Comparison of biochemical parameters

**Fig. 2:**
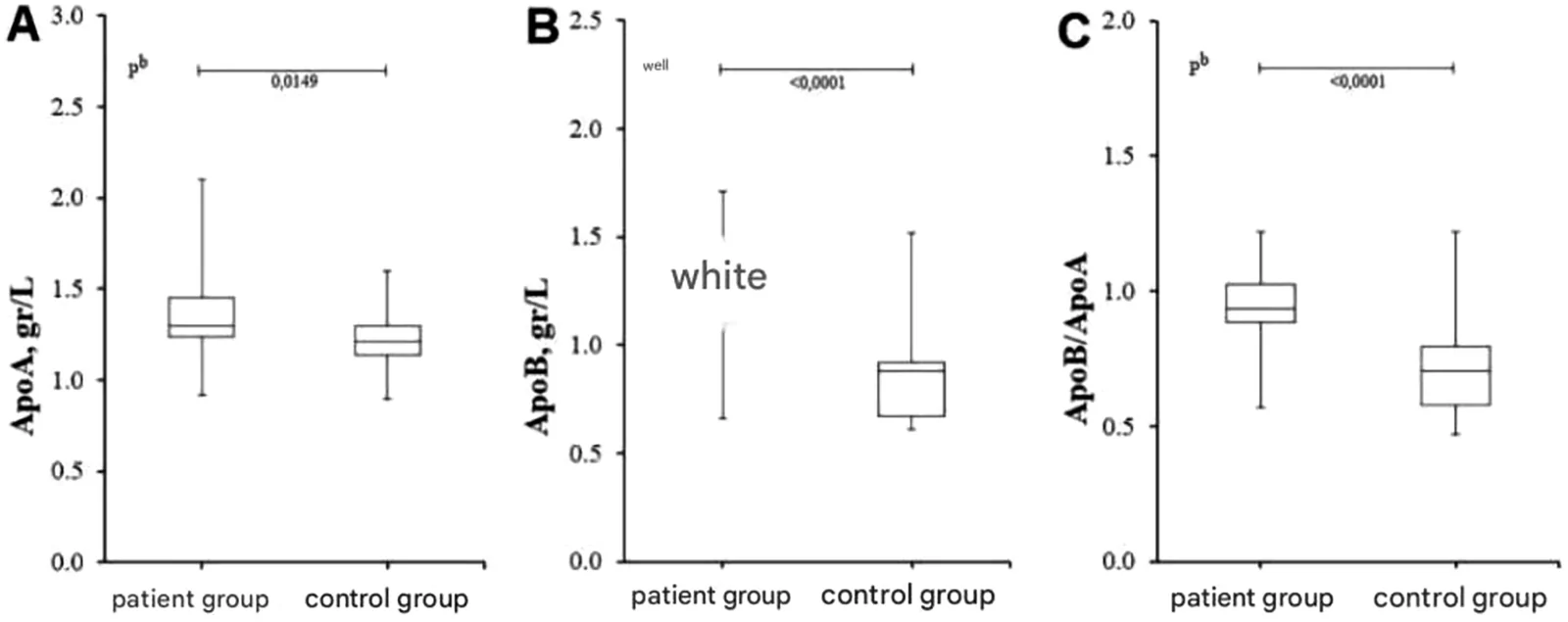
Box-plot graph representing ApoA, ApoB and ApoB/ApoA levels of normal and hypertensive patient groups. It is seen that ApoA, ApoB and ApoB/ApoA values are higher in the hypertensive patient group. **A** Mann Whitney U test, **B** Unpaired t test. **C** Illustrates the relationship between serum sortilin concentration andcardiovascular risk indicators in the study population.

When the groups were evaluated in terms of lipid profiles, cholesterol, LDL, ApoA and ApoB levels were statistically higher in the HT patient group (*p* < 0.01) (Figs. 1A,B and 2A,B). Similarly, ApoB/ApoA values, which are a calculation parameter, were also found to be higher in the HT patient group (*p* < 0.0001) (Fig. 2C and Table 2).

**Table 2:** Comparison of biochemical parameters of groups.

	HT patient group (A)	Control group (B)	*p* value
N	55	65	–
Cholesterol, mmol/L	5.79 ± 1.38 5.73 (2.89–9.65)	4.10 ± 1.08 4.01 (2.58–7.15)	< 0.0001^a^*
Triglyceride, mmol/L	3.09 ± 2.75 2.24 (0.44–15.04)	2.80 ± 0.80 2.82 (0.91–4.69)	0.1644^a^
LDL, mmol/L	2.92 ± 1.09 2.86 (0.50–4.83)	2.28 ± 0.72 2.10 (1.20–4.21)	0.0005^a^*
VLDL, mmol/L	1.4 ± 1.3 1.0 (0.2–6.8)	1.2 ± 0.4 1.2 (0.4–2.1)	0.4512^a^
ApoA, gr/L	1.34 ± 0.22	1.24 ± 0.20	0.0083^b^*
ApoB, gr/L	1.26 ± 0.23 1.28 (0.66–1.71)	0.88 ± 0.22 0.88 (0.61–1.52)	< 0.0001^a^*
ApoB/ApoA	0.96 ± 0.20	0.73 ± 0.22	< 0.0001^b^*

### Comparison of preinflammatory biomarker and sortilin levels

**Fig. 3:**
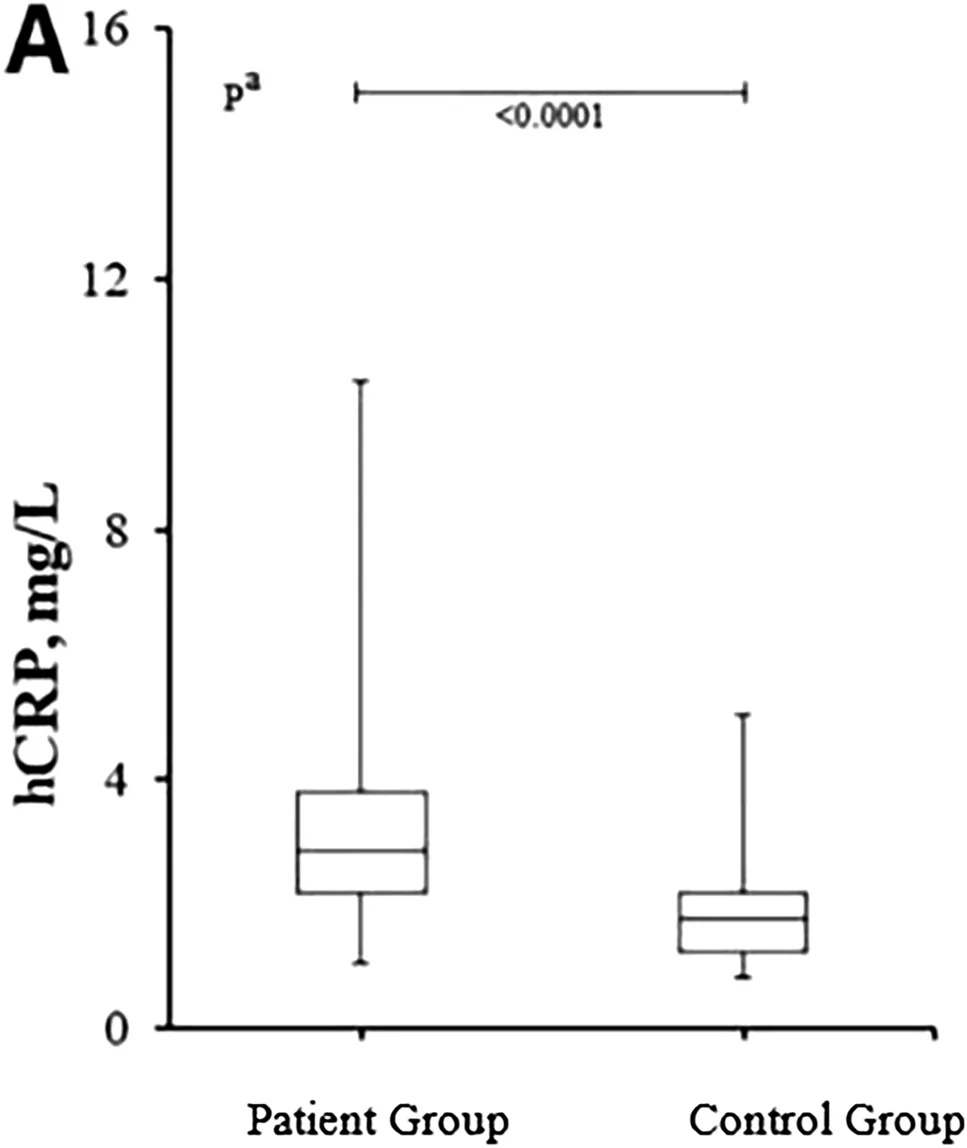
Box-plot graph representing hs-CRP and sortilin levels in normotensive and hypertensive groups. hs-CRP and sortilin levels were higher in the hypertensive group. **A** Mann–Whitney U test.

When the groups were examined in terms of inflammatory biomarkers (Table 3), the hs-CRP of the HT patient group, and sortilin were found to be statistically significantly higher compared to the control group (Fig. 3A,B).

**Table 3:** Comparison of inflammatory biomarker and sortilin values of groups.

	HT patient group (A)	Control group (B)	*p* value
n	55	65	–
Hs-CRP, mg/L	3.1 ± 1.7	1.7 ± 0.8	< 0.0001^b^*
Sortilin, ng/mL	2.2 ± 0.9 2.1 (0.6–5.5)	1.6 ± 0.7 1.4 (0.8–4.6)	< 0.0001^a^*

*Statistical significance level *p* < 0.05. Nonparametric data are given as mean ± SD and median (min–max), parametric data are given as mean ± SD. SD: Standard deviation, hs-CRP: high sensitive CRP-reactive protein.

^a^Mann–Whitney U Test.

^b^Unpaired t test.

In addition, a statistically significant positive correlation was observed between serum sortilin levels and the ApoB/ApoA ratio in the overall study population (Spearman r = 0.42, 95% CI 0.26–0.56; *p* < 0.001). This association remained significant when hypertensive and normotensive groups were analyzed separately, indicating a consistent relationship between sortilin and lipid atherogenicity independent of blood pressure status.

According to the correlation matrix analysis results (Table 4), while no correlation was detected between DBP and the available parameters, a low to moderate positive correlation was observed between SBP values and cholesterol, LDL, ApoB, ApoB/ApoA, hs-CRP and sortilin levels.

**Table 4:** Correlation matrix analysis between blood pressure, lipid profiles and sortilin parameters of all study groups.

	A:	B:	C:	D:	E:	F:	G:	H:	I:	J:	K:	L:	M:
A: SBP	1.0000												
B: DBP	− 0.0245	1.0000											
C: Chol	**0.5275**	− 0.1668	1.0000										
D: TG	0.0742	− 0.0310	**0.3026**	1.0000									
E: LDL	**0.3073**	0.0614	**0.4058**	0.1397	1.0000								
F: VLDL	0.0919	− 0.0514	**0.3215**	**0.9800**	0.1130	1.0000							
G: ApoA	0.2058	− 0.0155	0.2468	− 0.0951	0.1333	− 0.0462	1.0000						
H: ApoB	**0.5703**	− 0.1824	**0.7573**	0.1719	**0.3220**	0.1868	0.2591	1.0000					
I: ApoB/A	**0.4817**	− 0.1766	**0.5520**	0.0598	0.2665	0.0467	− 0.1013	**0.7949**	1.0000				
K: Sortilin	**0.4419**	− 0.0424	0.2827	0.0508	0.1920	0.0564	0.0096	0.2477	0.2059	0.2739	1.0000		
M: hCRP	**0.3702**	− 0.0601	**0.3804**	0.0867	0.2528	0.0880	− 0.0174	**0.3798**	**0.3390**	0.2559	0.1731	**0.4016**	1.0000

Significant values are in [bold].

**Fig. 4:**
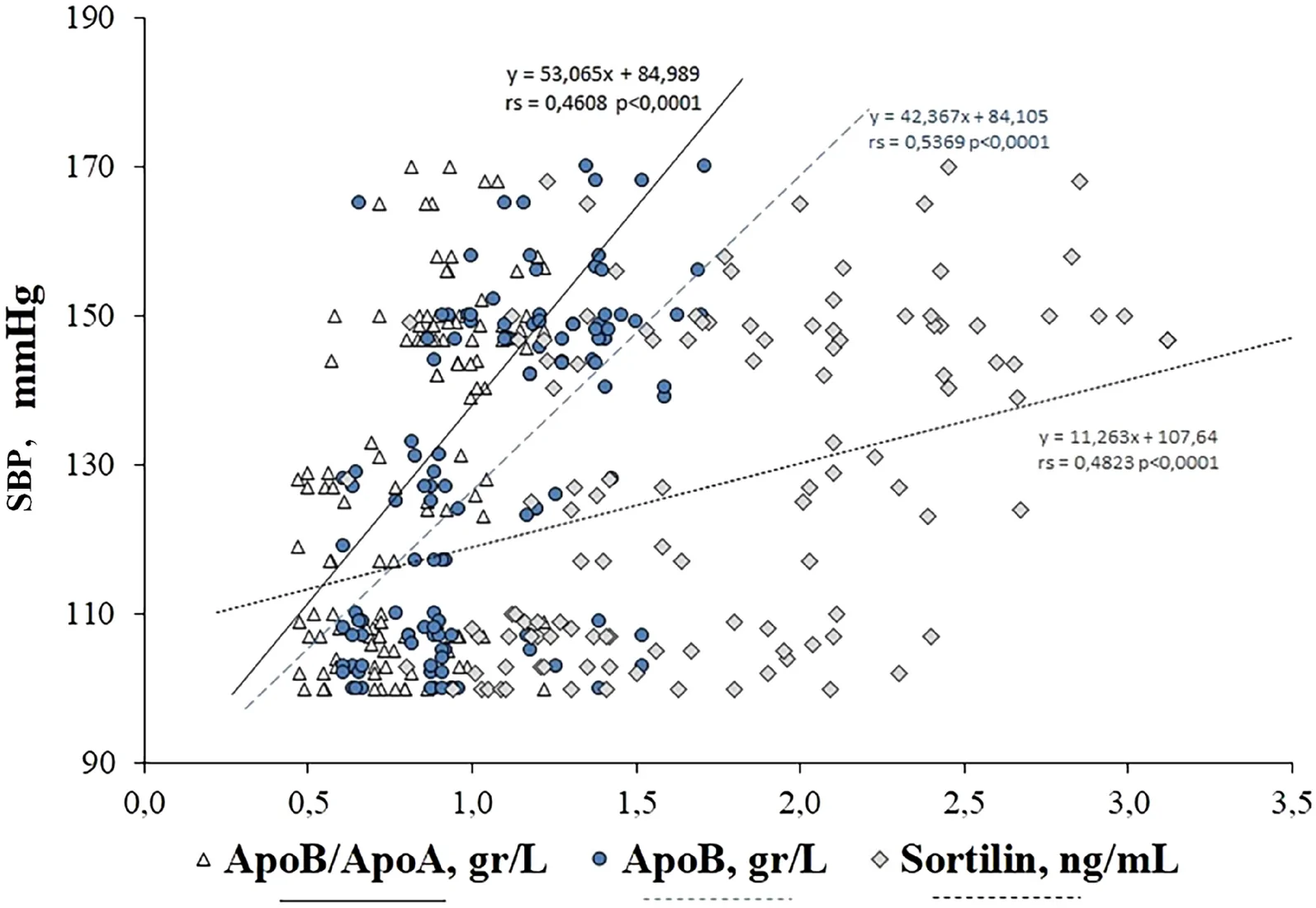
Spearman correlation graph representing the relationship between SBP and ApoA, ApoB and ApoB/ApoA levels in all case groups. SBP values show the highest correlation with ApoB levels. rs: Spearman correlation.

There was a moderate positive correlation between SBP and ApoB and ApoB/ApoA levels (rs = 0.5369, 95% CI 0.3912–0.6564; *p* < 0.0001 and Spearman r = 0.4608, 95% CI 0.3020–0.5947; *p* < 0.0001, respectively) (Fig. 4).

**Fig. 5:**
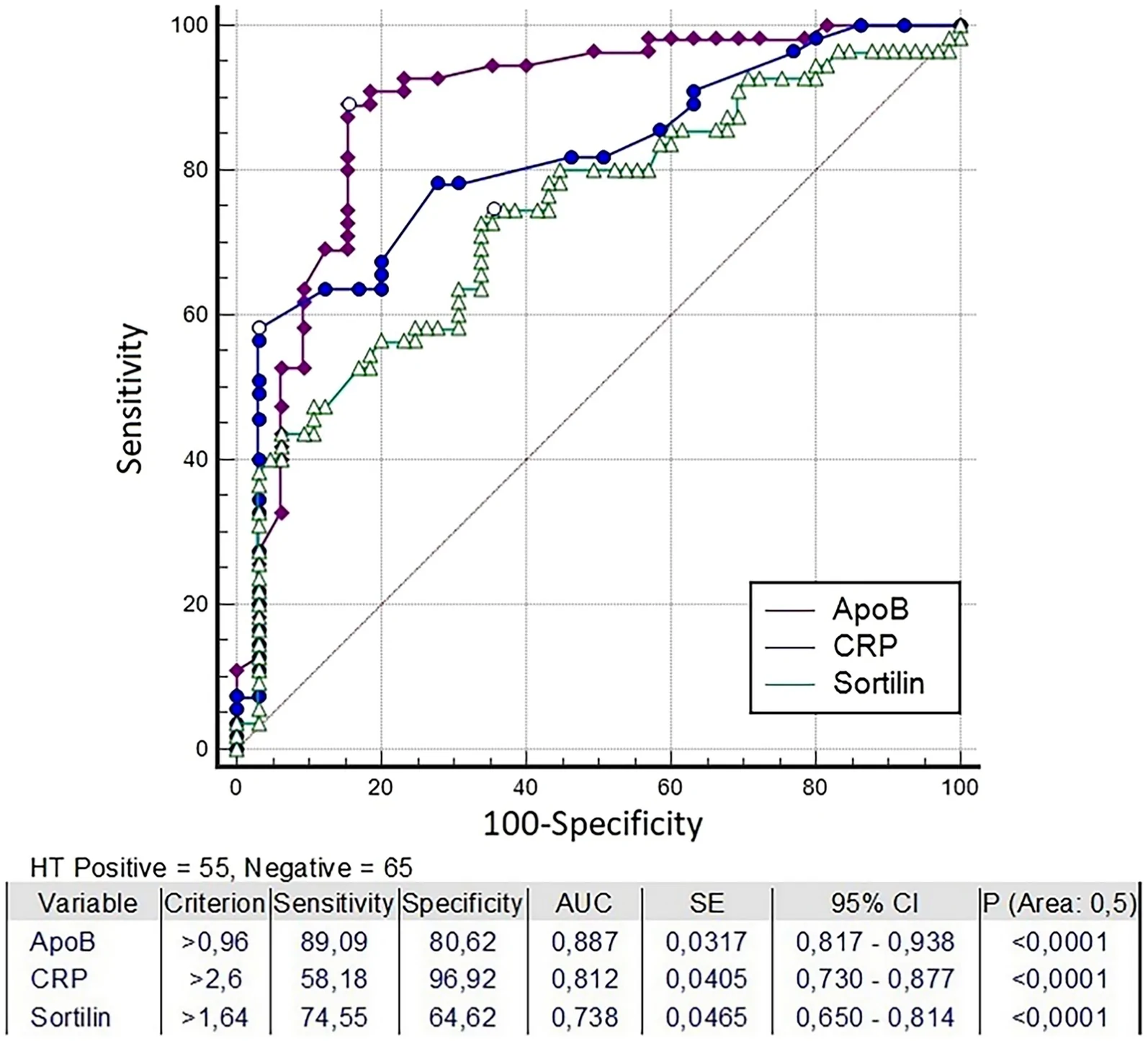
ROC analysis results to determine the discriminatory performance of ApoB, hs-CRP and sortilin tests in detecting hypertension status. ApoB and sortilin are seen to have good discriminatory performance, respectively.

In the ROC analysis performed to evaluate the discriminatory ability of ApoB, CRP and sortilin values in determining HT, the best cut-off value of the ApoB test (> 0.96 g/L) was found to have sensitivity of 89.09%, specificity of 80.62% and AUC of 0.887 (*p* < 0.0001) (Fig. 5). The best cut-off value of the hs-CRP test (> 2.6 mg/dL) was found to have sensitivity of 58.18%, specificity of 96.92% and AUC of 0.812 (*p* < 0.0001).The best cut-off value of the sortilin test (> 1.64 ng/mL) was found to have sensitivity of 74.55%, specificity of 64.62% and AUC (Area Under The Curve) of 0.738 (*p* < 0.0001).

Overall, the results of statistical analyses (group comparisons, correlation analysis, regression, and ROC analysis) suggest that ApoB, hs-CRP, and sortilin are associated with hypertension and may be related to cardiovascular risk.

In addition, the combined use of these biomarkers may improve the discrimination of hypertension status compared with individual markers.

Therefore, these findings suggest that sortilin, together with hs-CRP and ApoB, may be associated with the disease status of patients with hypertension.

## Discussion

Since hypertension is a multifactorial disease, it also requires investigation using various laboratory diagnostic methods due to the presence of many factors in its pathogenesis^11–13^. It should be noted that various methods and criteria are used in the diagnosis of hypertension, but simple laboratory parameters that can confirm these criteria are still needed^1,13^.

By now, it seems established that sortilin plays a part in the development of dyslipidemia and associated diseases, e.g., atherosclerosis. The question is in what way. Findings in separate strains of knockout mice strongly suggest that sortilin deficiency is protective and lowers the level of circulating LDL-C and cholesterol. In humans, however, low levels of sortilin (in carriers of the major haplotype/allele) are paralleled by an increased risk of developing hyperlipidemia and disease^14–16^. Sortilin is a signaling and protein transport receptor that plays an important role in various cellular functions. The main ligands of sortilin are neurotensin, progranulin, and lipoprotein lipase^17–20^. Its main functions include intracellular transport and sorting of proteins, in particular, cellular signaling that triggers biological reactions^16,21^. It promotes accurate transport and incorporation of proteins into target cellular sites in the Golgi apparatus and endosomal system^19,22^. It also plays an important role in endocytosis in the cell. Namely, when sortilin binds to its ligands, the resulting ligand-receptor complexes enter the cell and the process of endocytosis is initiated. In this way, signals or molecules coming from outside the cell are transmitted into the cell^10,23^. It regulates lipid metabolism by acting on the enzyme lipoprotein lipase and is involved in the uptake and metabolism of lipids in the cell. These various functions of sortilin are important for maintaining cellular order and homeostasis. Thus, the study of sortilin is important for understanding cellular metabolism and pathological processes, and possibly for developing treatments^8,9^. Sortilin is associated with many diseases and pathologies. Among them, it is associated with neurodegenerative diseases such as Alzheimer’s disease, cancers such as breast cancer and pancreatic cancer, metabolic disorders such as metabolic syndrome, obesity and type 2 diabetes, as well as cardiovascular pathologies and hypertension due to the development of atherosclerosis^22,24,25^. However, no definitive conclusion has been made regarding the use of sortilin in the diagnosis of cardiovascular pathology and hypertension. In light of the above information, this study investigated the possibility of using sortilin alone or in combination with other biomarkers as a prognostic marker for understanding and diagnosing the pathophysiology of hypertension, the most common disease today.

The study conducted among young and middle-aged (23–45 years) male military personnel compared the SBP and DBP values in individuals diagnosed with hypertension with healthy individuals and revealed that only the SBP values differed in individuals diagnosed with hypertension, which suggests that pathologies affecting the processes associated with vascular tone are more pronounced in this group of patients. In addition, the higher level of sortilin in the AH group confirms our opinion. Again, higher levels of cholesterol, ApoB and LDL, which accelerate atherosclerosis of the vascular structure and affect systolic blood pressure, were found in the AG group and were associated with the protein sortilin, which plays an important role in lipid metabolism. The results of the study conducted by Chu and colleagues (Chu X) also confirm our findings. Researchers have reported that sortilin, which plays a role in glucose and lipid metabolism, is associated with hypertension, coronary heart disease, and carotid atherosclerosis^1,13,26^. Again, some researchers have reported an association between inflammatory markers such as hs-CRP and hypertension, which is consistent with our study^20,27^. However, there are also studies reporting no difference between hs-CRP (or other inflammatory markers) and hypertension^18,19^. The possible reason for this may be due to the characteristics of the selected patient population or the lack of exclusion criteria. In our study, the detection of significant correlations between SBP and hs-CRP, cholesterol, ApoB, ApoB/ApoA, and sortilin was considered as evidence that hypertension occurs as a manifestation of a complex relationship between lipid metabolism, inflammation, and sortilin.

In addition, the significant correlation of sortilin values with cholesterol, ApoB/ApoA indicates the existence of this complex relationship. In other words, many factors play a role in the development of hypertension, and the impact of each factor on the development of hypertension will be different. In this study, the finding that high levels of sortilin, ApoB and CRP increase the risk of hypertension confirms our prediction above. Most of the parameters used in the diagnosis of diseases are not sufficient evidence on their own. Therefore, several additional auxiliary parameters are needed. The best evidence for this was the results of the logistic regression analysis for the assessment of arterial hypertension. It was found that ApoB and sortilin together explained 79% of the prediction of hypertension status. This finding indicates that sortilin or ApoB markers alone may not be sufficient. Moreover, in ROC analyses, no parameter achieved sufficient diagnostic power to be used alone.

### Limitations

This study has several limitations. First, its cross-sectional design precludes causal inference. Second, the relatively small sample size and the inclusion of only male military personnel may limit the generalizability of the findings. Third, potential confounding factors such as lifestyle, diet, and stress levels were not fully accounted for. Additionally, the lack of longitudinal follow-up limits the ability to assess predictive value over time.

## Conclusion

In this cross-sectional study, sortilin, ApoB, and hs-CRP were found to be associated with hypertension in military personnel. These findings suggest a potential link between lipid metabolism, inflammation, and blood pressure regulation. However, due to the observational and cross-sectional design of the study, causal relationships cannot be established.

Moreover, the relatively small sample size and the inclusion of only male military personnel may limit the generalizability of the findings. Therefore, these biomarkers should be considered as potential, rather than definitive, indicators associated with cardiovascular risk.

Further large-scale and prospective studies are required to better clarify the role of these biomarkers in the pathophysiology and risk stratification of hypertension.

## Data Availability

The data that support the findings of this study are available from the corresponding author upon reasonable request. Due to ethical restrictions and confidentiality regarding military personnel, the data are not publicly available.
